# Significant prognostic values of differentially expressed-aberrantly methylated hub genes in breast cancer

**DOI:** 10.7150/jca.33433

**Published:** 2019-10-21

**Authors:** Lina Qi, Biting Zhou, Jiani Chen, Wangxiong Hu, Rui Bai, Chenyang Ye, Xingyue Weng, Shu Zheng

**Affiliations:** 1 Cancer Institute (Key Laboratory of Cancer Prevention and Intervention, China National Ministry of Education), 2nd Affiliated Hospital, School of Medicine, Zhejiang University, Hangzhou, Zhejiang, 310009, China.; 2 Research Center for Air Pollution and Health, School of Medicine, Zhejiang University, Hangzhou, Zhejiang, 310009, China.; 3 Department of Surgical Oncology, The Second Affiliated Hospital of Zhejiang University School of Medicine, Hangzhou, Zhejiang, 310016, China.

**Keywords:** Breast cancer, Expression, Methylation, Prognosis, Bioinformatics

## Abstract

**Introduction**: Abnormal status of gene expression plays an important role in tumorigenesis, progression and metastasis of breast cancer. Mechanisms of gene silence or activation were varied. Methylation of genes may contribute to alteration of gene expression. This study aimed to identify differentially expressed hub genes which may be regulated by DNA methylation and evaluate their prognostic value in breast cancer by bioinformatic analysis.

**Methods**: GEO2R was used to obtain expression microarray data from GSE54002, GSE65194 and methylation microarray data from GSE20713, GSE32393. Differentially expressed-aberrantly methylated genes were identified by FunRich. Biological function and pathway enrichment analysis were conducted by DAVID. PPI network was constructed by STRING and hub genes was sorted by Cytoscape. Expression and DNA methylation of hub genes was validated by UALCAN and MethHC. Clinical outcome analysis of hub genes was performed by Kaplan Meier-plotter database for breast cancer. IHC was performed to analyze protein levels of EXO1 and Kaplan-Meier was used for survival analysis.

**Results**: 677 upregulated-hypomethylated and 361 downregulated-hypermethylated genes were obtained from GSE54002, GSE65194, GSE20713 and GSE32393 by GEO2R and FunRich. The most significant biological process, cellular component, molecular function enriched and pathway for upregulated-hypomethylated genes were viral process, cytoplasm, protein binding and cell cycle respectively. For downregulated-hypermethylated genes, the result was peptidyl-tyrosine phosphorylation, plasma membrane, transmembrane receptor protein tyrosine kinase activity and Rap1 signaling pathway (All p< 0.05). 12 hub genes (TOP2A, MAD2L1, FEN1, EPRS, EXO1, MCM4, PTTG1, RRM2, PSMD14, CDKN3, H2AFZ, CCNE2) were sorted from 677 upregulated-hypomethylated genes. 4 hub genes (EGFR, FGF2, BCL2, PIK3R1) were sorted from 361 downregulated-hypermethylated genes. Differential expression of 16 hub genes was validated in UALCAN database (p<0.05). 7 in 12 upregulated-hypomethylated and 2 in 4 downregulated-hypermethylated hub genes were confirmed to be significantly hypomethylated or hypermethylated in breast cancer using MethHC database (p<0.05). Finally, 12 upregulated hub genes (TOP2A, MAD2L1, FEN1, EPRS, EXO1, MCM4, PTTG1, RRM2, PSMD14, CDKN3, H2AFZ, CCNE2) and 3 downregulated genes (FGF2, BCL2, PIK3R1) contributed to significant unfavorable clinical outcome in breast cancer (p<0.05). High expression level of EXO1 protein was significantly associated with poor OS in breast cancer patients (p=0.03).

**Conclusion**: Overexpression of TOP2A, MAD2L1, FEN1, EPRS, EXO1, MCM4, PTTG1, RRM2, PSMD14, CDKN3, H2AFZ, CCNE2 and downregulation of FGF2, BCL2, PIK3R1 might serve as diagnosis and poor prognosis biomarkers in breast cancer by more research validation. EXO1 was identified as an individual unfavorable prognostic factor. Methylation might be one of the major causes leading to abnormal expression of those genes. Functional analysis and pathway enrichment analysis of those genes would provide novel ideas for breast cancer research.

## Introduction

Breast cancer is the most frequently diagnosed cancer among females worldwide following lung cancer 1. Aberrant gene expression plays an important role in tumorigenesis, progression and metastasis of breast cancer and it is considered to be the consequence of not only genetic defects (such as TP53, PIK3CA mutation, BRCA1/BRCA2 inactivation, Cyclin D1 amplification 2) but also epigenetic modifications 3. Epigenetic alterations in breast cancer consist of DNA methylation, RNA methylation, histone modification , non-coding RNAs (especially miRNA and lncRNA) regulation and so no 4. This study focused on DNA methylation, one of the most widely studied epigenetic modifications. DNA methylation occurs with the addition of a methyl (CH3) group from S-adenosylmethionine (SAM) into cytosine residues of the DNA template 5, mostly located on cytosine-phosphate-guanine (CpGs) dinucleotides. Both DNA hypermethylation and hypomethylation can be involved in diverse processes of breast cancer development and prognosis 6.

In clinical practice, though breast cancer is classified into three subtypes according to hormone receptor status, growth factor receptor status and Ki-67 which reflected partial prognostic information. And serum CA 15-3, CEA level, BRCA1/2 mutation status, PALB2 mutation status and circulating tumor DNA methylation might provide additional information for prognosis. However, heterogeneity of prognosis still exists. Therefore, more biomarkers are still urgently needed for more accurate prognosis. To date, there are many public databases for gene expression and methylation whose data was provided by published researches. Among them, plenty of researches have demonstrated the correlation between DNA methylation and prognosis of breast cancer, but the comprehensive profile and the interaction network of these aberrantly-expressed methylated genes still remain elusive. This study was aimed to identify aberrantly expressed hub genes that might be regulated by DNA methylation in breast cancer and to evaluate the prognostic value of these genes by using public databases. Several accessible software, databases, simple operations and basic bioinformatic knowledge were needed to complete this study and results might provide directions for further research.

## Materials and Methods

### Microarray and RNASeq data

In the initiation of present study, we screened the breast cancer expression microarray and methylation microarray datasets in GEO DataSets of NCBI (https://www.ncbi.nlm.nih.gov/gds/),sorted by sample number (From high to low). Study type was restricted to expression profiling by array and methylation profiling by array, and datasets both including breast cancer and normal breast samples were utilized. Finally, expression microarray datasets GSE54002, GSE65194 and methylation microarray datasets GSE20713, GSE32393 were included. 16 normal breast tissues and 200 primary breast cancer tissues were selected in GSE54002. 11 normal breast tissues and 130 primary breast cancer tissues were enrolled in GSE65194 (Platform: GPL570, Affymetrix Human Genome U133 Plus 2.0 Array). For methylation microarray dataset, GSE20713 enrolled 12 normal breast tissues and 234 primary breast cancer tissues. GSE32393 enrolled 23 normal breast tissues and 114 invasive breast tumor tissues. (Platform: GPL8490, Illumina HumanMethylation27 Bead Chip. HumanMethylation27_270596_v.1.2). For prognostic evaluation, GSE 65194 and GSE43568 datasets were included. 104 primary breast cancer tissues were enrolled in GSE43568 (Platform: GPL570, Affymetrix Human Genome U133 Plus 2.0 Array). As for RNASeq data, all normal and matched breast tumor level 3 mRNA expression HiSeq data sets (RNASeqV2) was obtained from The Cancer Genome Atlas (TCGA) (October 2015).

### Screening for upregulated-hypomethylated and downregulated-hypermethylated genes

GEO2R, an online analyzing tool of GEO DataSets, was utilized to analyze differentially expressed genes (DEG) between breast cancer tissues and normal tissues in expression microarray datasets and differential methylation in methylation microarray datasets. P value < 0.05 and |t| >2 were used as cutoff criteria to identify differential expression and methylation genes. Next, FunRich software (Functional Enrichment analysis tool, latest version 3.1.3 was download from http://www.funrich.org/) 7 was used to identify overlapping genes from GSE54002, GSE65194, GSE20713 and GSE32393. Finally, the overlapping genes were identified as upregulated-hypomethylated and downregulated-hypermethylated genes in breast cancer from the previous four datasets.

### GO and KEGG pathway enrichment analysis

DAVID (The Database for Annotation, Visualization and Integrated Discovery, https://david.ncifcrf.gov/), an online analysis tool box consists of an integrated biological knowledgebase and analytic tools aimed at systematically extracting biological meaning from large gene/protein lists 8, was used for gene functional and pathway enrichment analysis. The gene ontology (GO) analysis 9 consisted of analysis of biological process (BP), cellular component (CC), molecular function (MF) and Kyoto Encyclopedia of Genes and Genomes (KEGG) pathway enrichment analysis 10 were performed for the identified downregulated-promotor hypermethylated genes. The top 5 GO analysis terms and top 10 KEGG pathways were visualized in the result. P value < 0.05 was used as statistical significance.

### Construction of protein-protein interaction (PPI) network and identification of hub genes

STRING (Search Tool for the Retrieval of Interacting Genes, https://string-db.org/), an online protein interaction analysis tool, was performed to construct PPI network of the previous identified downregulated-promotor hypermethylated genes. Homo sapiens were selected as the organism for subsequent analysis. Medium confidence 0.4 of Interaction score was regarded as the cut-off criterion for network visualization and disconnected nodes was hidden. Subsequently, Cytoscape software (latest version 3.7.0, download from http://www.cytoscape.org/), a network data integration, analysis and visualization tool, was conducted to identify hub genes and modules within PPI network. Degree >25 was used as cutoff criteria for hub gene identification. MCODE score >3 and number of nodes >4 were utilized as cutoff criteria for module identification.

### Validation of gene expression in UALCAN database

UALCAN (http://ualcan.path.uab.edu/), an online cancer transcriptome database, is designed to provide easy access to publicly available cancer transcriptome data (TCGA and MET500 transcriptome sequencing) 11. This database was used to compare expression level of hub genes between normal breast tissue and primary invasive breast carcinoma.

### Validation of gene methylation in MethHC database

MethHC (http://MethHC.mbc.nctu.edu.tw), a database of DNA Methylation and gene expression in Human Cancer, integrates data from TCGA (The Cancer Genome Atlas), which includes 18 human cancers in more than 6000 samples, 6548 microarrays and 12 567 RNA sequencing data. Differential DNA methylation was compared by average beta value in tumor sample and matched normal samples in different genes 12.

### Survival analysis of genes in Kaplan Meier-plotter database

Clinical outcome analysis of hub genes in breast cancer was performed by Kaplan Meier-plotter database 13 whose background database was established using gene expression data and survival information of 1,809 patients downloaded from GEO (Affymetrix HGU133A and HGU133+2 microarrays). Overall survival analysis of 16 hub genes was performed separately in its default settings with 300 months follow-up.

### COX regression analysis and Kaplan Meier-plotter construction

IBM SPSS statistics 22 was used for cox regression analysis. Relative ratio (RR) and 95% CI were used for statistical analysis. After univariable analysis, hub genes were enrolled in multivariable analysis whose p value <0.1. Backward regression was used for multivariable regression analysis. Finally, hub genes included in the last step was selected as combined genes for prognostic analysis. Next, the upper 50% gene expression patient was defined as high expression group and lower 50% gene expression was defined as low expression group. Exp(B) from multivariable analysis was saved for the following classification. The upper 50% RR was defined as high risk group and lower 50% was defined as low risk group. ROC curve analysis was performed for single gene and combine genes group for predicting 5-year overall survival. Kaplan Meier-plotter analysis was performed for specific group identified previously. Log Rank (Mantel-Cox) was used for statistical analysis.

### Tissue microarrays (TMAs) and IHC staining

TMAs were purchased from Outdo Biotech (Shanghai, China), and contained 140 breast cancer samples. But 7 samples were missing after we finished IHC staining. Clinicopathological characteristics of these 133 samples are listed in Table S2. This experiment was approved by the Ethics Committee of the Second Affiliated Hospital, Zhejiang University School of Medicine. Primary antibodies against EXO1 (Huabio, ER1908-42, China) (1:200) was used for IHC staining.

EXO1 expression scores were blindly evaluated by two pathologists using the immunoreactivity score (IRS), based on the percentage of positive cells and the intensity of staining. When the two pathologists had a very different score, we asked a third pathologist to evaluate the slide. The percentage of positive cells was graded as follows: 0 (negative), 1 (<10%), 2 (10%-50%), 3 (51%-80%), 4 (>80%). The intensity of staining was graded as follows: 0 (no color reaction), 1 (mild reaction), 2 (moderate reaction), 3 (intense reaction). IRS was multiplied by the two scores. In this study, EXO1 expression was defined as low (IRS ≤6) or high (IRS >6).

## Results

### Identification of abnormal expressed-methylated genes in breast cancer

**Figure 1:**
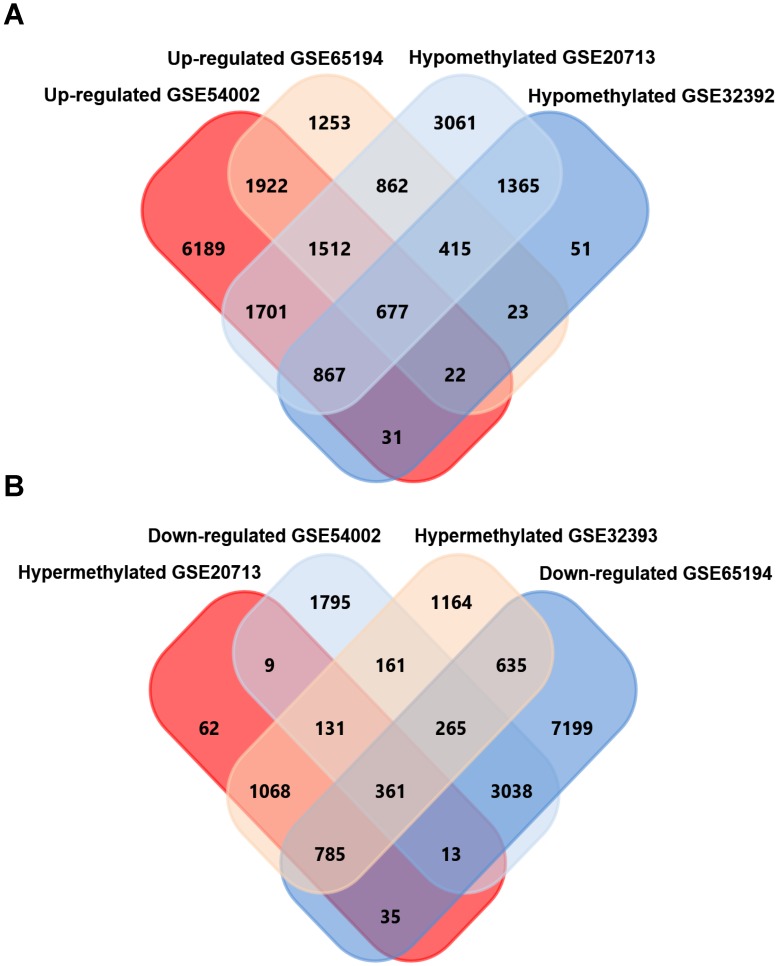
Screening of upregulated-hypomethylated and downregulated-hypermethylated genes in four GSE datasets (Expression microarray datasets GSE54002, GSE65194 and methylation datasets GSE20713, GSE32393).

GEO2R, an online analyzing tool of GEO DataSets, was utilized to screen differentially expressed genes in expression microarray (GSE54002, GSE65194), and differentially methylated genes in methylation microarray (GSE20713, GSE32393), separately. As a result, 9945 upregulated, 4857 downregulated genes from GSE54002 and 6074 upregulated, 8714 downregulated genes from GSE65194 were identified. For methylation microarray data, 2075 hypermethylation, 8282 hypomethylation genes from GSE20713 and 3752 hypermethylation, 2750 hypomethylation genes from GSE32393 were identified. 677 upregulated-hypomethylation genes (Fig. 1A) and 361 downregulated-hypermethylation genes (Fig. 1B) were sorted out by overlapping genes from four GSE datasets using FunRich software. These 1038 genes were identified as aberrantly expressed-methylated genes in breast cancer.

### GO functional enrichment analysis

Functional enrichment analysis of 677 upregulated-hypomethylation genes and 361 downregulated-hypermethylation genes was performed by GO analysis. The result was listed in Table 1. The top 5 terms of analytical result including biological process, cellular component and molecular function, were visualized in the table ranking by P-value from low to high (p<0.05). The most significant biological process, cellular component and molecular function enriched in the 677 upregulated-hypomethylation genes was viral process, cytoplasm and protein binding, separately. For downregulated-hypermethylated genes, the result was peptidyl-tyrosine phosphorylation, plasma membrane, transmembrane receptor and protein tyrosine kinase activity, separately.

### KEGG pathway enrichment analysis

The top 10 KEGG pathways enriched by DAVID were demonstrated in Table 2 by P-value from low to high (p<0.05). The most significant pathway enriched in 677 upregulated-hypomethylation genes was Pathways in cell cycle, including 16 genes (YWHAZ, E2F5, ANAPC13, DBF4, TTK, CDK6, PTTG1, CDC27, MCM4, MCM5, CCNE2, CCND1, CDKN2A, MCM7, MAD2L1, BUB3). For 361 downregulated-hypermethylation genes, the most significant pathway enriched was Rap1 signaling pathway, including 16 genes (EGFR, FGFR1, MAGI2, FGF7, FLT1, PGF, MRAS, GRIN2A, KIT, CALML3, PDGFRA, RAP1A, GNAS, FGF2, INSR, PIK3R1). Famous pathways P53, PI3K-Akt, and Ras signaling pathway which play important roles in life process were also enriched in KEGG pathway analysis.

### PPI network construction and hub gene validation

PPI network was constructed by STRING database and hub gene validation was identified by Cytoscape software. Module analysis was performed by MCODE, an application in Cytoscape software. PPI network was displayed in Fig. S1 and Fig.S2. Different color of nodes and edges meat for different known status for proteins and different protein-protein association strength. Five modules for upregulated-hypomethylated genes (Fig. S1B-F) and 3 modules for downregulated-hypermethylated genes (Fig. S2B-D) were identified in the PPI network according to the cutoff of MCODE score >3 and number of nodes >4. 16 hub gene were identified by the cutoff criteria of degree >25 (Table. 3), 12 genes for upregulated-hypomethylated and 4 genes for downregulated-hypermethylated genes group.

### Expression validation of the hub genes in TCGA dataset through UALCAN database

**Figure 2:**
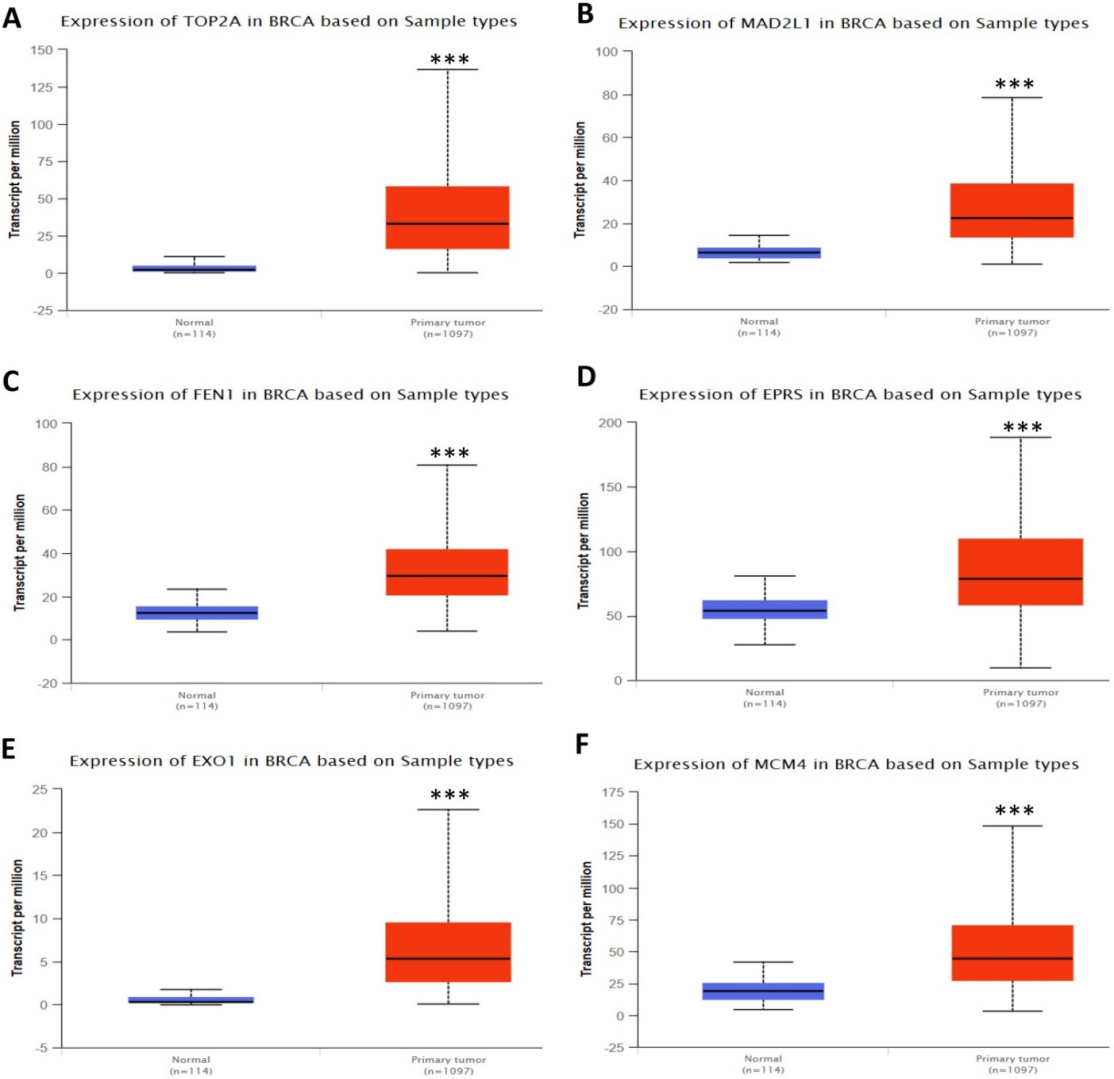
Expression validation in UALCAN database for upregulated-hypomethylated hub genes (Data from TCGA database). A: TOP2A; B: MAD2L1; C: FEN1; D: EPRS; E: EXO1; F: MCM4. ***: p<0.001.

**Figure 3:**
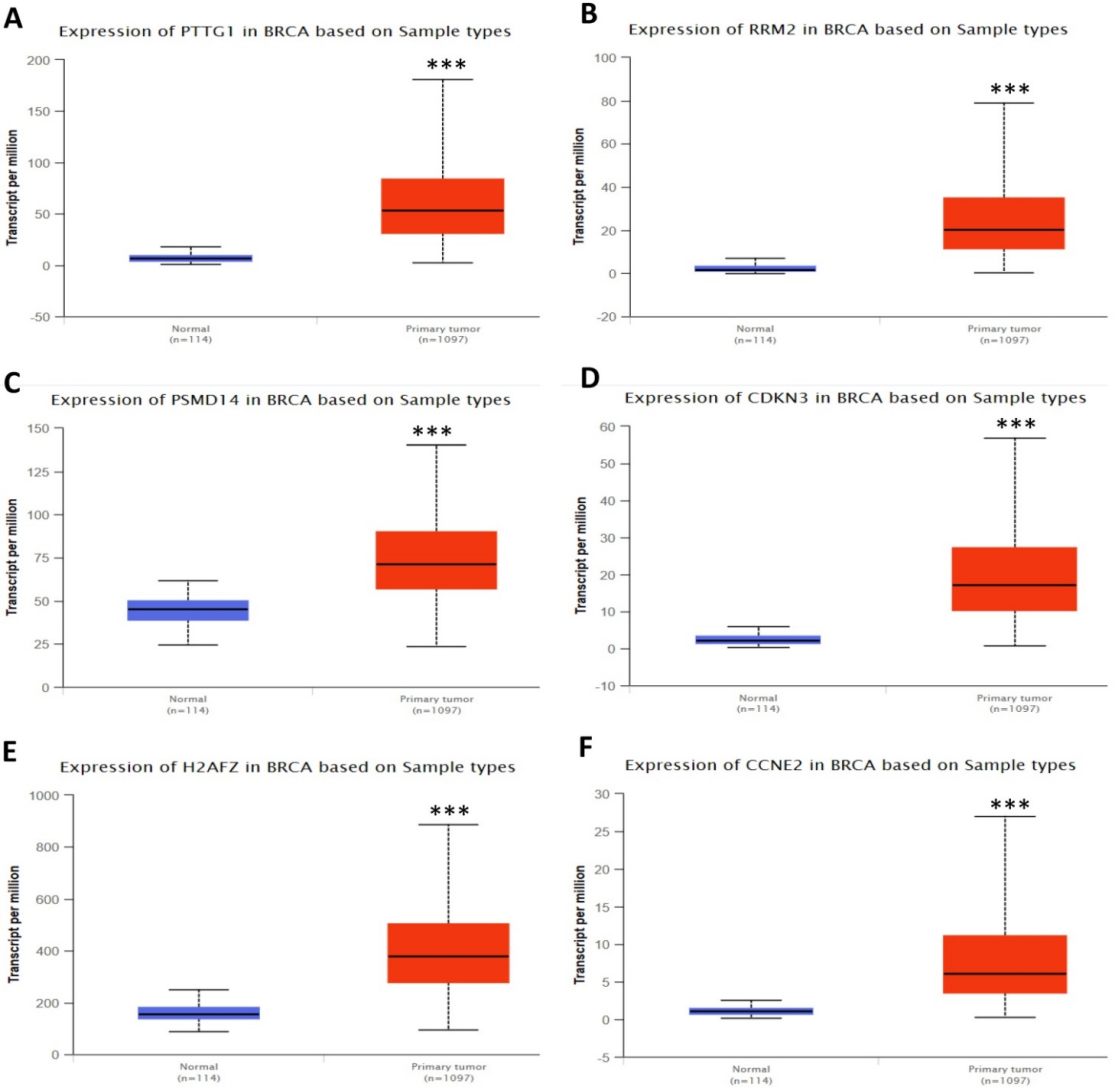
Expression validation in UALCAN database for upregulated-hypomethylated hub genes (Data from TCGA database). A: PTTG1; B: RRM2; C: PSMD14; D: CDKN3; E: H2AFZ; F: CCNE2. ***: p<0.001.

**Figure 4:**
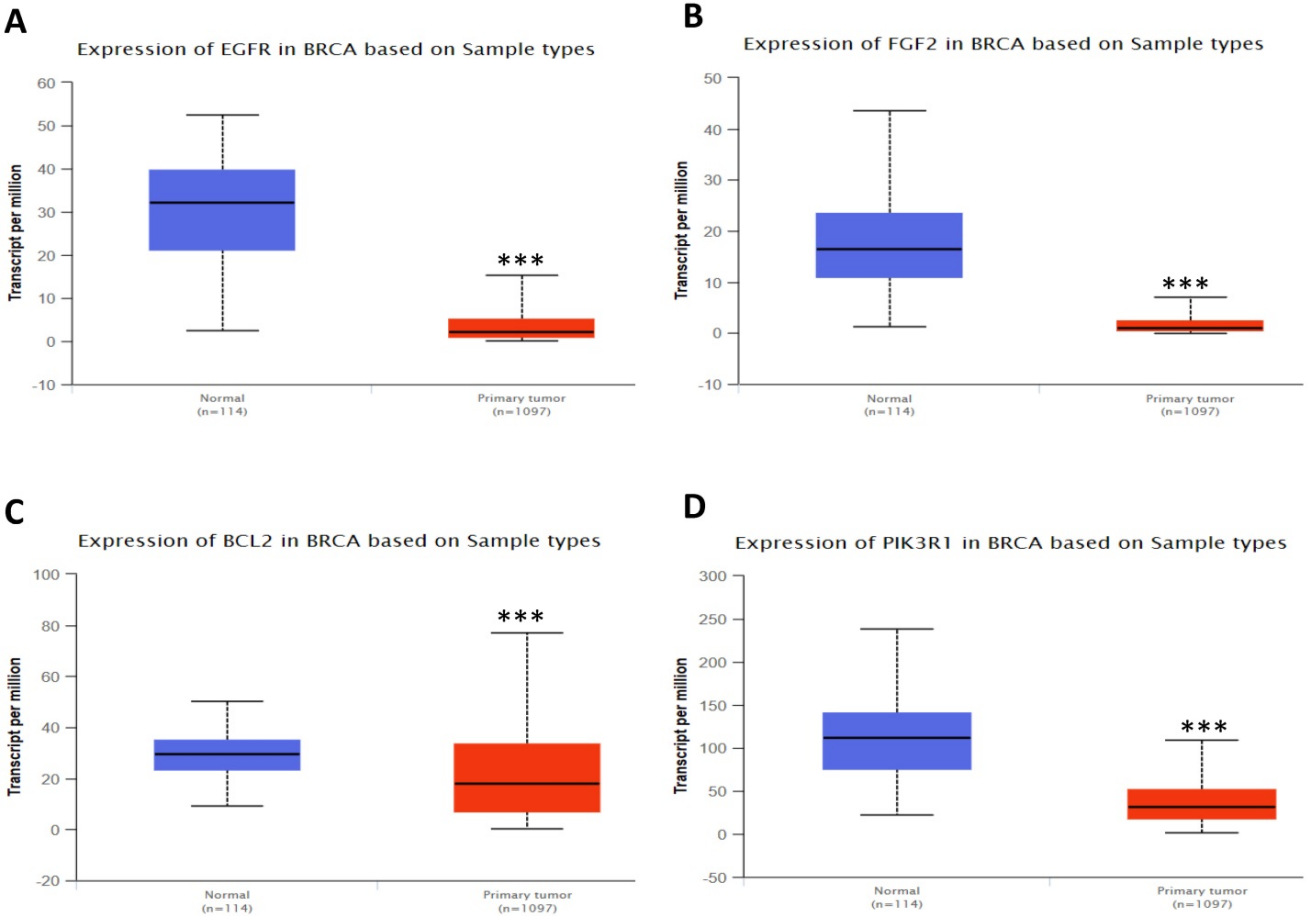
Expression validation in UALCAN database for downregulated-hypermethylated hub genes (Data from TCGA database). A: EGFR; B: FGF2; C: BCL2; D: PIK3R1. ***: p<0.001.

In order to validate the expression status of 16 hub genes in breast cancer compared to normal breast tissue, UALCAN database was utilized to confirmed the result. All of the 16 hub genes were found to be significantly differential expressed in invasive breast carcinoma. 12 upregulated hub genes (TOP2A, MAD2L1, FEN1, EPRS, EXO1, MCM4, PTTG1, RRM2, PSMD14, CDKN3, H2AFZ, CCNE2) identified from GSE database were confirmed to be high expression genes in breast cancer (Fig. 2 and 3) (p<0.05) and 4 downregulated hub genes (EGFR, FGF2, BCL2, PIK3R1) confirmed to be low expression genes (Fig. 4) (p<0.05). P values for those genes were listed in Table. 3. What's more, we compared expression of 16 hub genes using breast cancer RNASeq data downloaded from TCGA and the result was consistent with the previous data (Fig. S3).

### Validation of DNA methylation in 16 hub genes in TCGA dataset through MethHC

**Figure 5:**
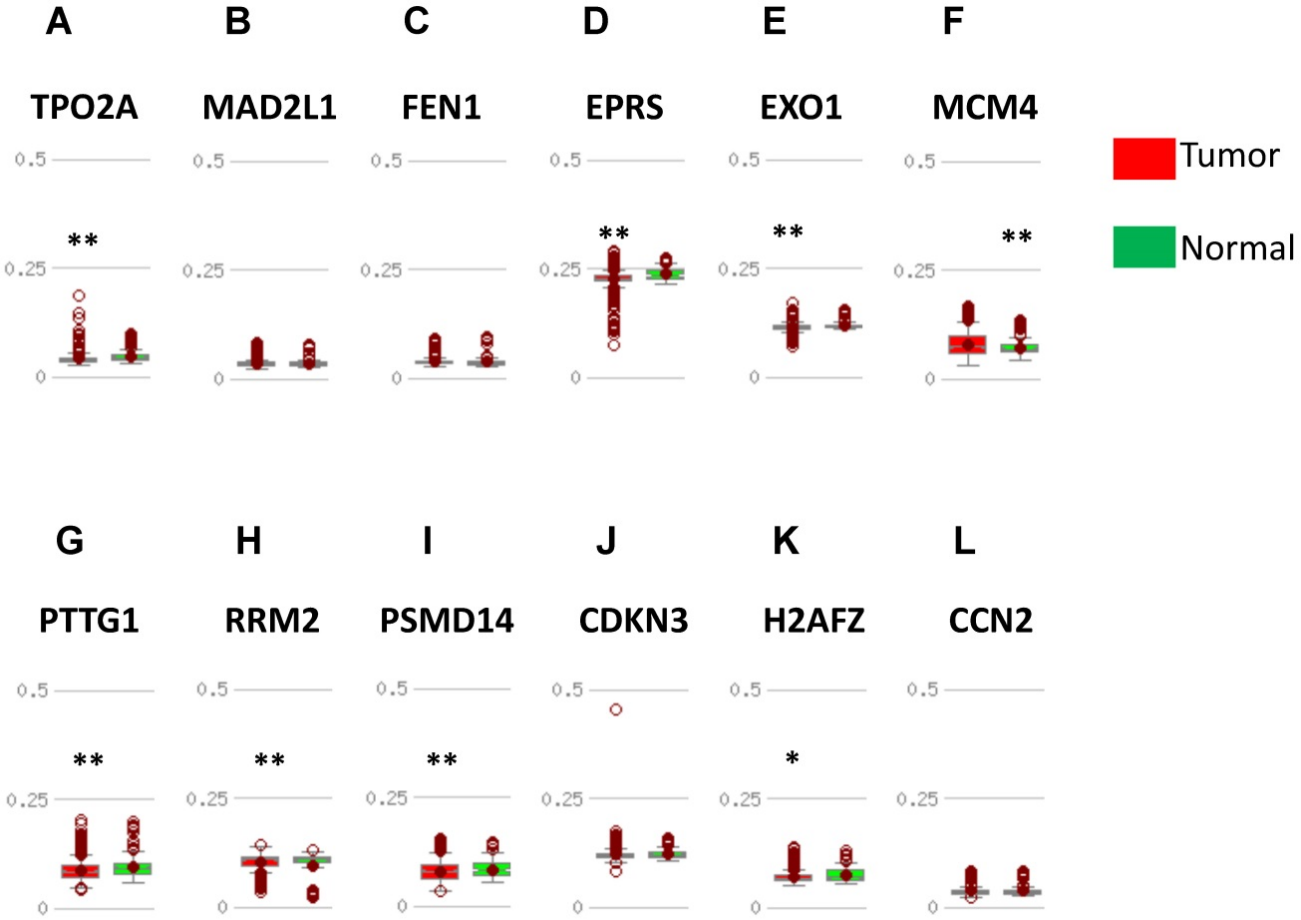
Validation of DNA methylation in upregulated hub genes in TCGA dataset through MethHC database. (A-L) Expression of upregulated-hypomethylated hub genes. A: TOP2A; B: MAD2L1; C: FEN1; D: EPRS; E: EXO1; F: MCM4, G: PTTG1; H: RRM2; I: PSMD14; J: CDKN3; K: H2AFZ; L: CCNE2. Green column: Normal breast tissue; Red column: invasive breast cancer. * p <0.05; ** p <0.005.

**Figure 6:**
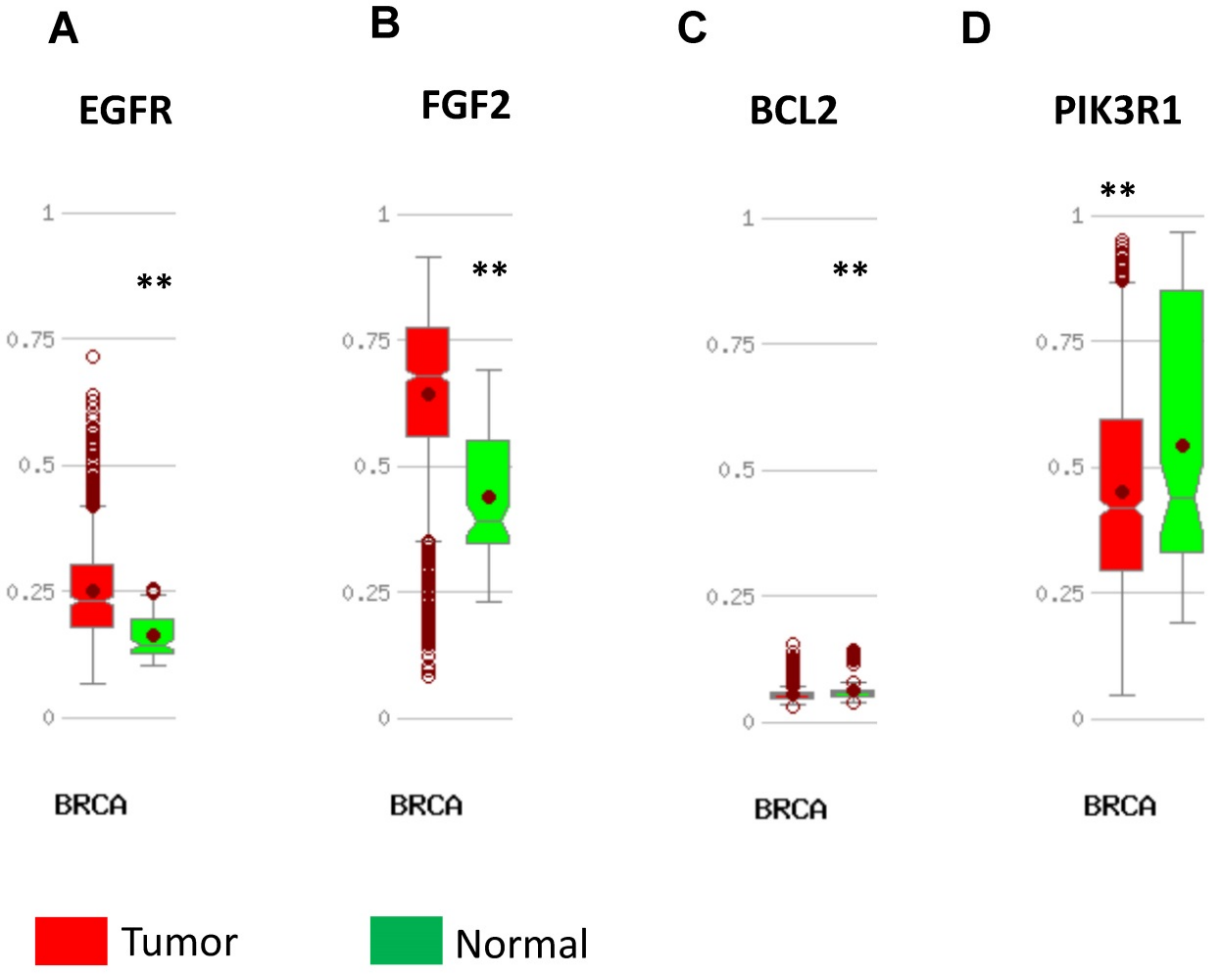
Validation of DNA methylation in downregulated hub genes in TCGA dataset through MethHC database. (A-D) A: EGFR; B: FGF2; C: BCL2; D: PIK3R1. Green column: Normal breast tissue; Red column: invasive breast cancer. * p <0.05; ** p <0.005.

DNA methylation status was validated in MethHC database. 7 in 12 upregulated hub genes (TOP2A, EPRS, EXO1, PTTG1, RRM2, PSMD14, H2AFZ) were found to be promoter hypomethylated (Fig. 5) and 2 in 4 downregulated genes (EGFR, FGF2) were validated to be promoter hypermethylated (Fig. 6). There was no significance of DNA promoter methylation between invasive breast cancer and normal breast tissues in 4 upregulated hub genes (MAD2L1, FEN1, CDKN3, CCN2) (Fig. 5). In contrast, MCM4 gene promoter was hypermethylated in breast cancer (Fig. 5) and BCL2, PIK3R1 gene promoter were hypomethylated (Fig. 6). DNA methylation status of hub genes and P values was listed in Table 3.

### Clinical outcome due to differential expression of the hub genes

**Figure 7:**
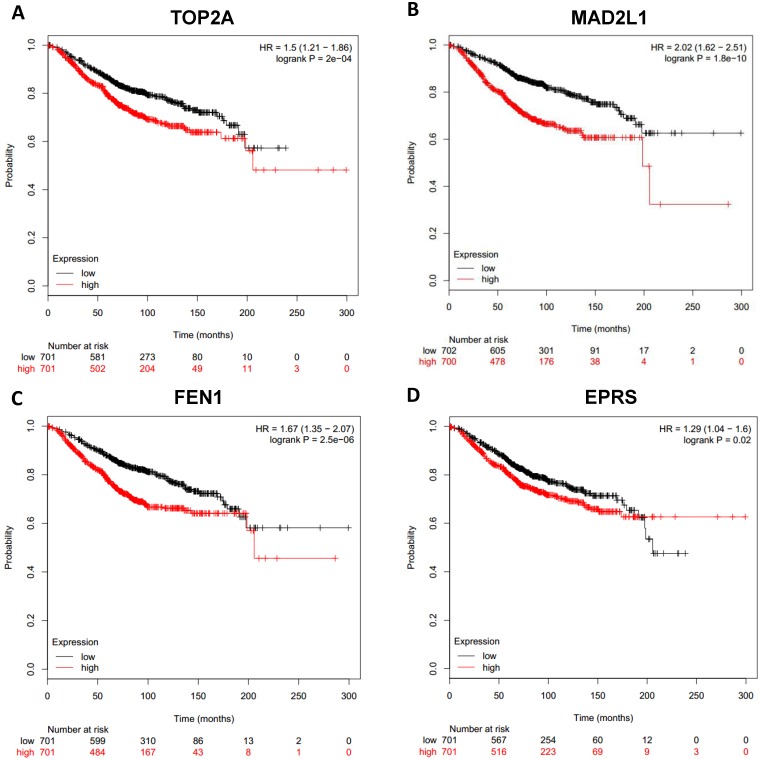
Prognostic values of upregulated hub genes in breast cancer (Kaplan Meier-plotter database). A: TOP2A; B: MAD2L1; C: FEN1; D: EPRS.

**Figure 8:**
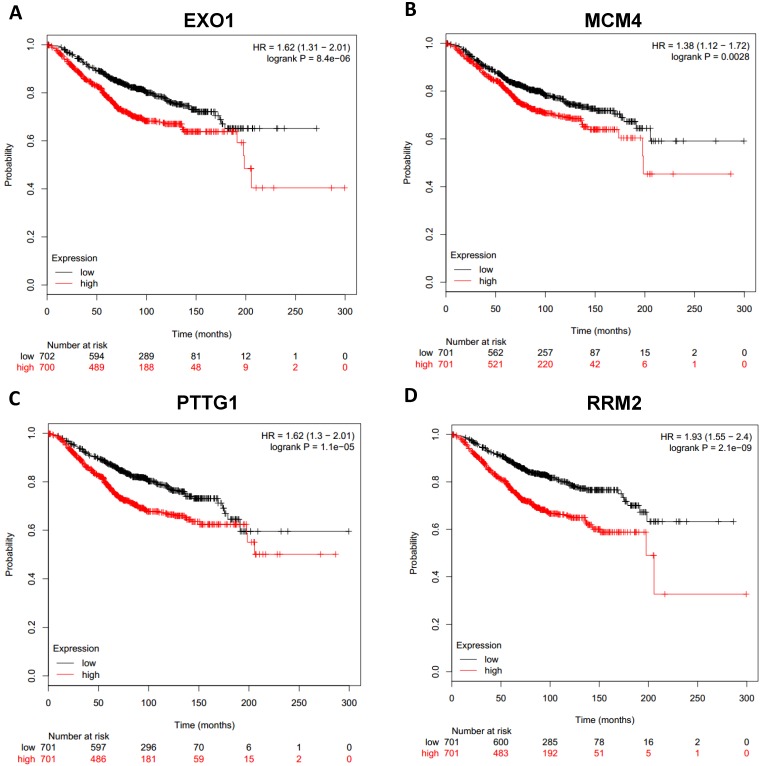
Prognostic values of upregulated hub genes in breast cancer (Kaplan Meier-plotter database). A: EXO1; B: MCM4; C: PTTG1; D: RRM2.

**Figure 9:**
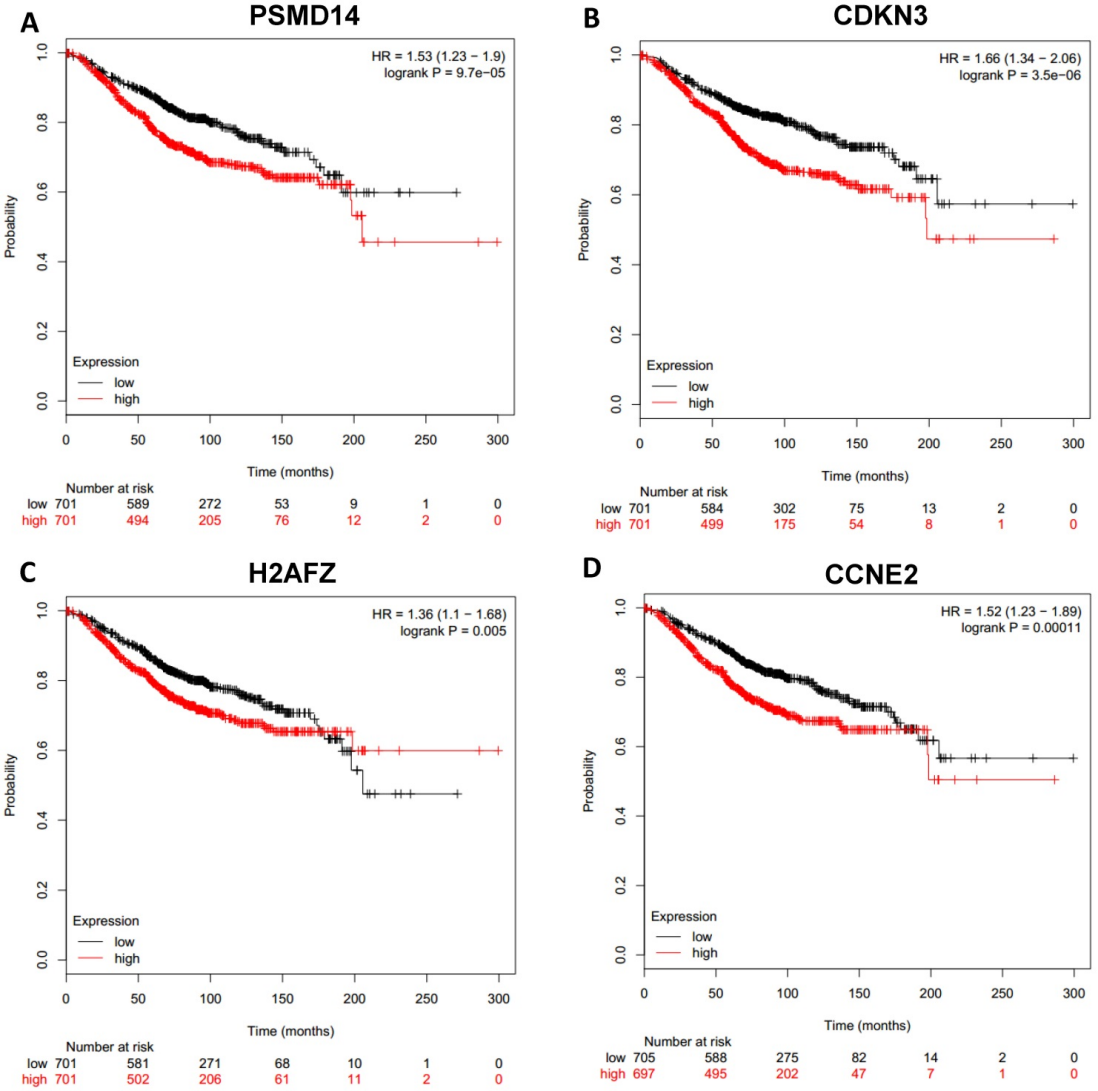
Prognostic values of upregulated hub genes in breast cancer (Kaplan Meier-plotter database). A: PSMD14; B: CDKN3; C: H2AFZ; D: CCNE2.

**Figure 10:**
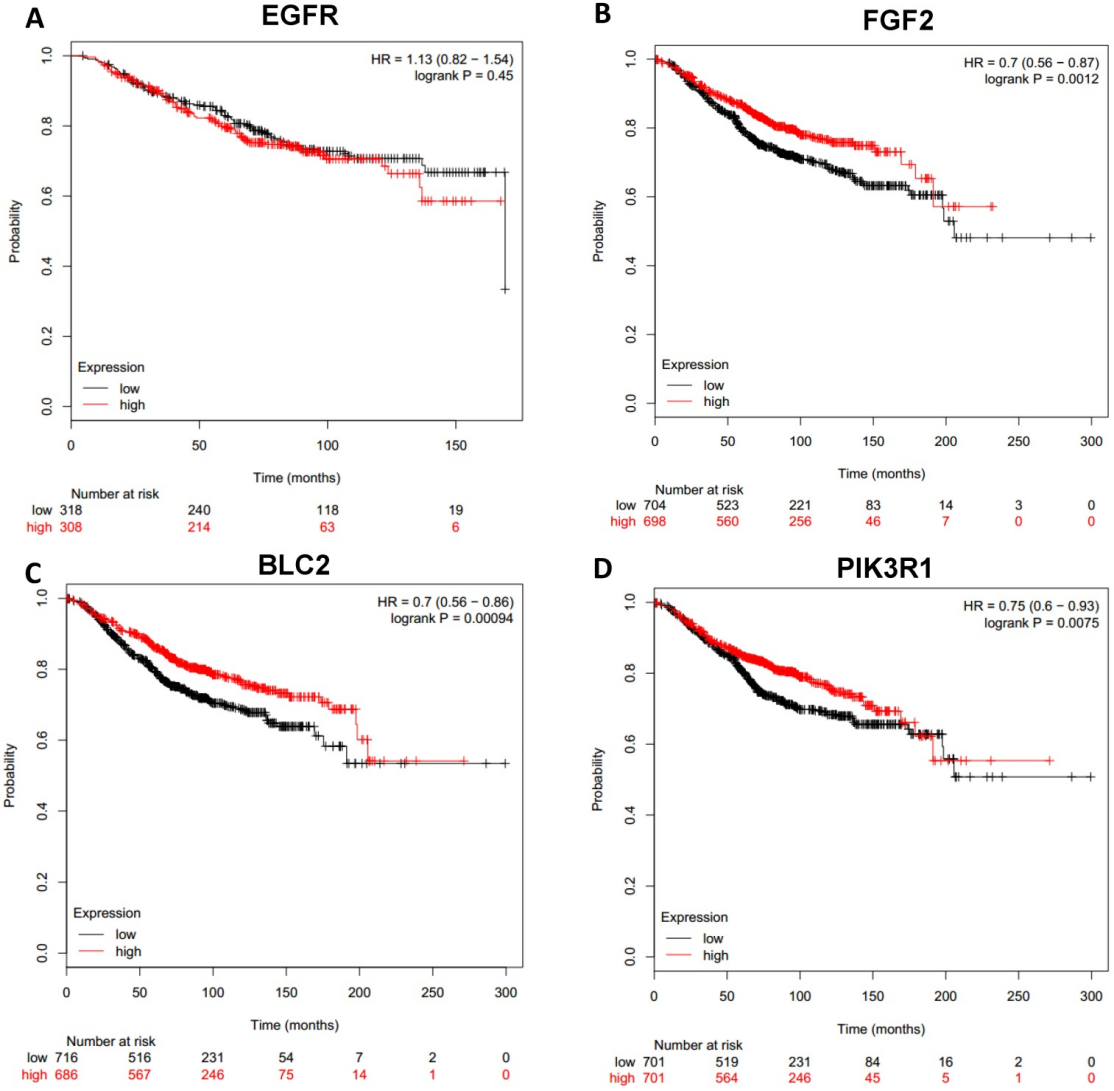
Prognostic values of downregulated hub genes in breast cancer (Kaplan Meier-plotter database). A: EGFR; B: FGF2; C: BCL2; D: PIK3R1.

To analyze prognostic value of 16 differentially expressed-methylated hub genes of breast cancer, Kaplan Meier-plotter database restricted to breast cancer was searched. Upregulation of TOP2A, MAD2L1, FEN1, EPRS (Fig. 7), EXO1, MCM4, PTTG1, RRM2 (Fig. 8), PSMD14, CDKN3, H2AFZ, CCNE2 (Fig. 9) and downregulation of FGF2, BCL2, PIK3R1 were significantly associated with poor overall survival, but downregulation of EGFR was not (Fig. 10). Among the 16 hub genes, upregulation of MAD2L1 was associated with unfavorable OS most significantly (HR=2.02 (1.62-2.51), P =1.8e-10).

What's more, we analyzed prognostic value of combined genes by using GSE42568 and GSE65194 datasets. Univariable cox regression analysis was performed first and we found that overexpression of MAD2L1, EXO1, MCM4, PTTG1 and CDKN3 was significantly associated with poor OS in GSE42568 dataset. Overexpression of EXO1, PTTG1, CCNE2 and PIK3R1 was significantly associated with poor OS in GSE65194 (Table S1) (p<0.05). TOP2A, MAD2L1, EXO1, MCM4, PTTG1, CDKN3 and CCNE2 in GSE42568 and TOP2A, FEN1, EXO1, MCM4, PTTG1, RRM2, CCNE2 in GSE65194 were enrolled in multivariable cox regression analysis whose p <0.1. Finally, EXO1, MCM4 and EXO1, PTTG1 were selected as combined gene group in GSE42568 and GSE65194 separately according to the final step of multivariable cox regression analysis. RR and 95% CI of univariable and multivariable cox analysis was listed in Table S1. Further, we classified patients into high expression and low expression group by single gene expression status. For combined gene group classification, we divided patients into two groups by relative ratio of multivariable analysis. ROC analysis for predicting 5-year overall survival was performed. AUC for combined group was equal to EXO1 in both datasets. Kaplan Meier-plotter was constructed for the previous identified groups. Prognostic value of combined EXO1 and MCM4 was no better than EXO1 in both datasets (Figure S4).

### Prognosis in patients with protein expression of EXO1

Since high EXO1 mRNA expression was identified as a strong individual prognostic factor in the previous part. Then, we performed IHC to investigated if EXO1 protein levels were significantly associated with OS in breast cancer patients.

**Figure 11:**
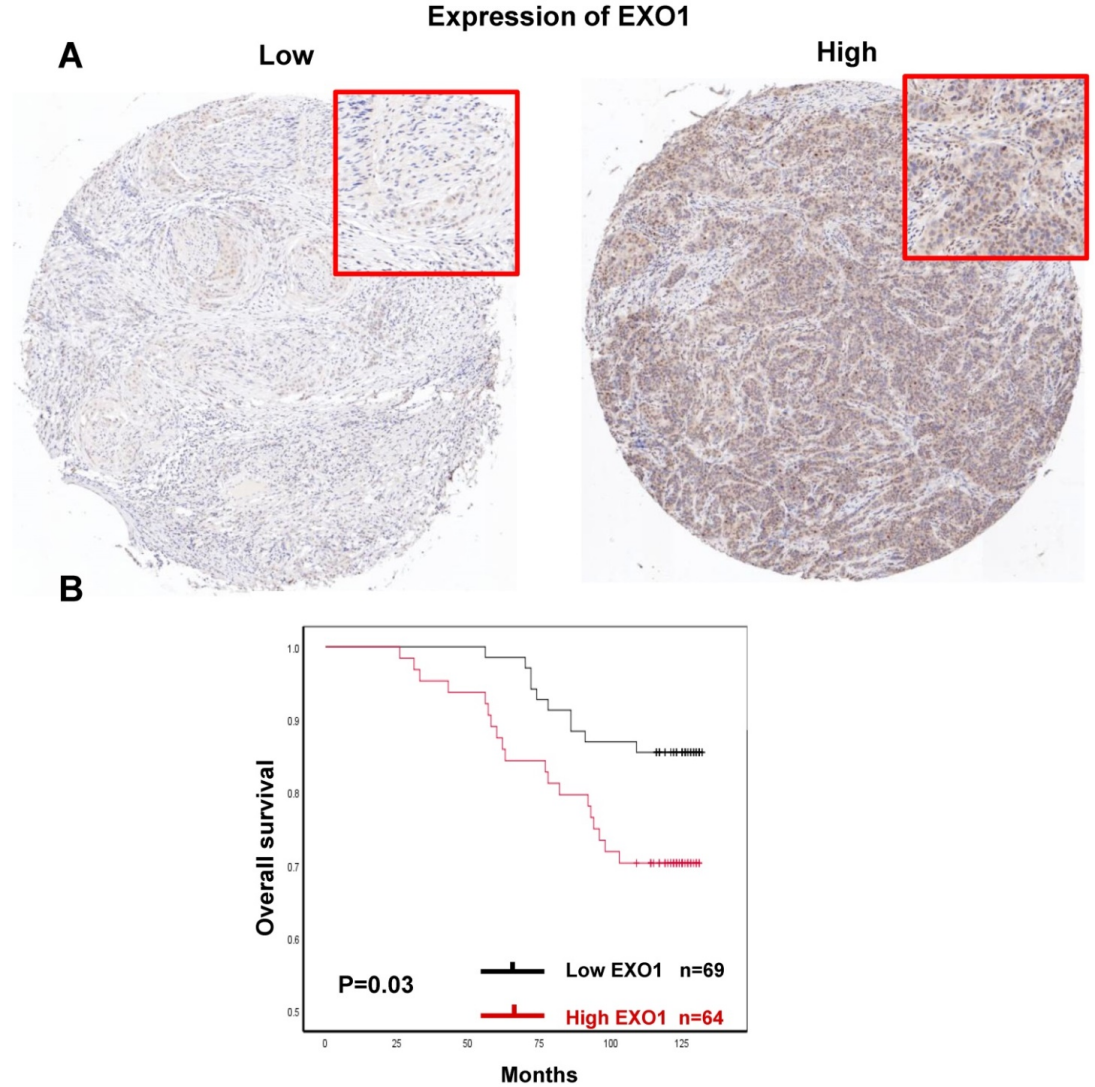
Prognostic significances of EXO1 protein expression in breast cancer. (A). Immunohistochemical analysis of EXO1 in breast cancer tissue with low staining and high staining; (B) Kaplan-Meier OS analysis of EXO1 protein expression in breast cancer patients. (Each tissue section was observed under microscopy with low magnification of 50× and high magnification of 100×.)

Finally, there were 133 of 140 tumor samples used for EXO1 protein expression analysis since 7 samples were missing in the process of IHC staining within the TMAs. Representative images of EXO1 low and high expression were shown (Fig.11A). EXO1 was mainly expressed in nuclear. 48.1% (64 of 133) patients exhibited EXO1 high expression (IRS >6). Median follow-up time was 123 months. For survival analysis, Kaplan-Meier analysis showed that high EXO1 protein expression was significantly associated with decreased OS in breast cancer patients (p=0.03, Fig. 11B). There were no differences in clinical Clinicopathological characteristics between EXO1 high expression and low expression cohorts (Table S2). The results suggested that in protein levels, high EXO1 expression was correlated with poor OS.

## Discussion

Recurrence and drug resistance are the main causes of mortality in breast cancer 14, therefore it is of great significance to evaluate the prognosis precisely and individually before progression. In this study, we discovered 677 upregulated-hypomethylated and 361 downregulated-hypermethylated genes from GSE54002, GSE65194, GSE20713 and GSE32393 by GEO2R and FunRich. Among them, 12 upregulated hub genes and 3 downregulated genes turn out to contribute to significant adverse clinical outcome in breast cancer.

As was suggested in KEGG pathway enrichment analysis by DAVID, MAD2L1, PTTG1, MCM4 and CCNE2 are both involved in cell cycle pathway. MAD2L1 is a mitotic spindle checkpoint gene. Among patients with primary breast cancer, higher expression of MAD2L1 and BUB1 existed in patients with ER-, PR-, and high-grade tumors compared to those with ER +, PR+, and low-grade tumors. High MAD2L1 expression was associated with poor overall survival 15, which is consistent with our results. PTTG1 is a regulator in chromosomal segregation. It can promote the proliferation of breast cancer cell through binding to P27 directly to induce nuclear exclusion of P27 16. MCM4 gene encodes a kind of minichromosomal maintenance proteins. MCM4 overexpression was found only weakly associated with shorter survival in breast cancer alone while the MCM complex had a better prognosis value 17. CCNE2 is known to promote G1-S transition. It has been demonstrated that CCNE2, targeted by miR-26a, miR-30b, might play an important role in acquired trastuzumab resistance in HER2+ breast cancer 18. TOP2A and RRM2 are both essential enzyme in DNA replication. TOP2A amplification often occurs with HER2 amplification 19. Previous researches have confirmed that upregulated TOP2A has unfavorable prognosis in breast cancer in both 5-year disease-free survival 20 and adjuvant treatment 21. High expression of RRM2 was associated significantly with decreased survival in all breast cancer subtypes and increased expression was shown in tamoxifen-resistant patients 22. Moreover, RRM2 can be targeted and suppressed by miR‐204‐5p and RRM2 overexpression can promote the proliferation and metastasis of breast cancer cells and suppressed cell apoptosis 23. FEN1 and CDKN3 are tumor suppressor genes while elevated expression of these genes may not seem to protect against carcinogenesis.

FEN1 is a DNA repair-specific nuclease. High level of FEN1 expression in breast cancer cells could reflect the enhanced proliferation or increased DNA damage of cancer cells 24. The level of FEN1 is inversely associated with cancer drug and radiation resistance 25. YY1^25^ and Nrf2 26 can down-regulated FEN1 expression through binding to the FEN1 promoter region and inactivating it. CDKN3 is involved in mitosis. Overexpression of CDKN3 predicts poor prognosis in cervical cancer 27 and lung adenocarcinoma 28 and it is also an effective biomarker in digestive system carcinomas 29, 30. However, there are not many relative researches about CDKN3 in breast cancer. EPRS is one of the Aminoacyl-tRNA synthetases, which are involved in protein translation. EPRS copy number gains in breast cancers tumors 31. Additionally, EPRS was selectively carbonylated in tumor tissue compared to matched adjacent healthy tissue in breast cancer 32. EXO1 is an exonuclease, which belongs to the mismatch repair system. EXO1 plays a role in replication fork degradation in BRCA1-and BRCA2-deficient cells 33. A meta-analysis of transcriptomic data of primary breast tumors also supports our finding about EXO1 34.

PSMD14 is a kind of deubiquitinating enzymes. There are few researches on PSMD14 in breast cancer. While recent studies have shown that high expression level of PSMD14 predicts poor prognosis of human esophageal squamous cell carcinoma 35. H2AFZ is an oncogenic histone variant that is expressed independently of DNA replication. SMYD3-mediated H2AFZ methylation accelerates G1-S transition and promotes breast cancer proliferation 36.

4 downregulated-hypermethylated hub genes (EGFR, FGF2, BCL2, PIK3R1) were sorted out by PPI network, and 3 of them turned out to be significantly associated with poor overall survival except EGFR. EGFR is a commonly mutant gene in many malignant tumors. EGFR is often overexpressed in breast cancer, especially in triple-negative breast cancer 37, while hypermethylation of EGFR can contribute to cetuximab resistance 38. FGF2 involves in cell proliferation and angiogenesis. FGF2 is hypermethylated in HR+ breast cancers and may have prognosis value 39 while it has low prognosis implication in triple-negative breast cancer 40. BCL2 belongs to the anti-apoptotic and anti-proliferative. A lot of studies have proved that positive BCL2 shows better prognosis in breast cancer 41-43. Therefore, we can infer that down-regulated expression of BCL2 predicts unfavorable prognosis.

PIK3R1 encodes the p85α regulatory subunit which regulates and stabilizes p110α. PIK3R1 has a lower frequency mutation than PIK3CA in breast cancer 2. Previous studies have confirmed that Low expression of PIK3R1 is associated with poor prognosis 44, 45.The prognosis value of some genes has been practiced in previous studies which is in accordance with our findings but more validation studies will still be needed. And the rest also needs to be explored in the future.

To the best of our knowledge, this is the first study to screen the differentially expressed-aberrantly methylated hub genes of significant prognosis value in breast cancer by simple bioinformatics. In this study, 4 microarray datasets were used to screen these genes to make the results much more convincing. Moreover, databases from TCGA dataset were used to validate the expression and DNA methylation of these 16 hub genes. In addition, the prognosis values of the 16 genes in breast cancer were analyzed through Kaplan Meier-plotter database. Except EFGR, other 15 differently-expressed genes are all significantly associated with prognosis. Also, the KEGG pathway analysis of these hub genes can provide guidance for further researches in breast cancer. What's more, we analyzed the prognostic value of combined genes and found that it was no better than EXO1 in both datasets. Not enough patients in either dataset or overexpression of EXO1 which was really a strong prognostic factor may cause this result. Protein levels of EXO1 were also analyzed and we found that high expression of EXO1 was associated with significant poor OS which suggested that EXO1 was a competitive prognostic factor for clinical application.

However, there were still some limitations in this study should be acknowledged. Though, expression of these genes were all remained to be significantly upregulated or downregulated after validation in UALCAN database, but only 7 of 12 hypomethylated and 2 of 4 hypermethylated genes was validated in another methylation database. Expression analysis may be more stable across different database. However, methylation status of some genes was varied among different platforms and databases. In our study, two methylation microarray datasets were performed on the same platform (Illumina HumanMethylation27 Bead Chip) in order to eliminate platform diversity and using another methylation database to make the result more convincing.

## Conclusion

In general, our study identified 9 aberrantly expressed-methylated hub genes in breast cancer significantly contributing to poor prognosis by bioinformatic analysis. Functional analysis and pathway enrichment analysis of those genes would provide novel ideas for further breast cancer research. 12 upregulated hub genes (TOP2A, MAD2L1, FEN1, EPRS, EXO1, MCM4, PTTG1, RRM2, PSMD14, CDKN3, H2AFZ, CCNE2) and 4 downregulated hub genes (EGFR, FGF2, BCL2, PIK3R1) might serve as diagnosis and poor prognosis biomarkers in breast cancer in the future by more research validation. Especially, EXO1 was identified as an individual unfavorable prognostic factor in this research.

## Supplementary Material

Supplementary figures and tables.

## Figures and Tables

**Table 1:** GO functional analysis of biological process, cell component and molecular function of differentially expressed-methylated genes.

Category	GO Analysis	Term	Count	%	P Value
**Hypomethylation and high expression**	GOTERM_BP_DIRECT	GO:0016032~viral process	32	0.03	4.25E-07
		GO:0009615~response to virus	16	0.01	1.75E-05
		GO:0032508~DNA duplex unwinding	10	0.01	3.36E-05
		GO:0032727~positive regulation of interferon-alpha production	6	0.01	4.94E-05
		GO:0006281~DNA repair	23	0.02	9.42E-05
	GOTERM_CC_DIRECT	GO:0005737~cytoplasm	278	0.26	1.27E-13
		GO:0005829~cytosol	196	0.18	4.83E-13
		GO:0016020~membrane	139	0.13	5.21E-11
		GO:0005654~nucleoplasm	163	0.15	2.50E-10
		GO:0070062~extracellular exosome	152	0.14	2.54E-07
	GOTERM_MF_DIRECT	GO:0005515~protein binding	451	0.41	3.08E-23
		GO:0004003~ATP-dependent DNA helicase activity	11	0.01	1.61E-07
		GO:0005524~ATP binding	89	0.08	1.51E-05
		GO:0044822~poly(A) RNA binding	68	0.06	1.35E-04
		GO:0042802~identical protein binding	47	0.04	7.65E-04
**Hypermethylation and low expression**	GOTERM_BP_DIRECT	GO:0018108~peptidyl-tyrosine phosphorylation	17	0.03	8.05E-08
		GO:0010863~positive regulation of phospholipase C activity	5	0.01	1.88E-05
		GO:0030335~positive regulation of cell migration	15	0.03	2.22E-05
		GO:0007568~aging	13	0.02	1.31E-04
		GO:0007155~cell adhesion	23	0.04	1.64E-04
	GOTERM_CC_DIRECT	GO:0005886~plasma membrane	119	0.21	7.89E-07
		GO:0005615~extracellular space	52	0.09	1.98E-06
		GO:0005829~cytosol	92	0.16	1.29E-04
		GO:0005925~focal adhesion	20	0.04	2.00E-04
		GO:0042383~sarcolemma	9	0.02	2.19E-04
	GOTERM_MF_DIRECT	GO:0004714~transmembrane receptor protein tyrosine kinase activity	9	0.02	6.30E-07
		GO:0004713~protein tyrosine kinase activity	14	0.02	2.39E-06
		GO:0046934~phosphatidylinositol-4,5-bisphosphate 3-kinase activity	9	0.02	2.88E-05
		GO:0043548~phosphatidylinositol 3-kinase binding	5	0.01	4.61E-04
		GO:0005088~Ras guanyl-nucleotide exchange factor activity	10	0.02	4.67E-04

**Table 2:** KEGG pathway enrichment analysis of differentially expressed-methylated genes.

Pathway ID	Pathway name	Gene Count	%	PValue	Genes
**Hypomethylation and high expression**					
hsa04110	Cell cycle	16	0.01	6.73E-04	YWHAZ, E2F5, ANAPC13, DBF4, TTK, CDK6, PTTG1, CDC27, MCM4, MCM5, CCNE2, CCND1, CDKN2A, MCM7, MAD2L1, BUB3
hsa03050	Proteasome	9	0.01	8.90E-04	PSMB5, PSMB4, PSMD14, PSMD12, PSMC2, SHFM1, PSMD1, PSMD4, PSMB9
hsa04115	p53 signaling pathway	10	0.01	0.00	CCNE2, PPM1D, CCND1, CDKN2A, RRM2, CYCS, CDK6, APAF1, THBS1, PERP
hsa03030	DNA replication	7	0.01	0.01	RFC3, MCM7, SSBP1, POLD1, MCM4, MCM5, FEN1
hsa04141	Protein processing in endoplasmic reticulum	16	0.01	0.01	RAD23B, DERL1, MAN1A2, NSFL1C, UBQLN1, ATF6, DNAJB11, FBXO6, DNAJB1, SAR1A, DNAJC1, SEC61A1, SSR2, SEC23B, SEC61G, SEL1L
hsa05152	Tuberculosis	16	0.01	0.02	RFX5, ATP6AP1, IL18, CEBPG, CYCS, ITGB2, ARHGEF12, ATP6V0B, CD74, VDR, MYD88, MAPK13, RIPK2, HSPD1, APAF1, FCGR3B
hsa03060	Protein export	5	0.00	0.02	SRPRB, SRP72, SRP9, SEC61A1, SEC61G
hsa05169	Epstein-Barr virus infection	12	0.01	0.03	DDX58, ICAM1, PSMD14, PSMD12, CD44, MAPK13, NFKBIE, PSMC2, SHFM1, PSMD1, PSMD4, TRAF5
hsa04623	Cytosolic DNA-sensing pathway	8	0.01	0.03	DDX58, POLR3K, POLR2K, IL18, IRF7, AIM2, CXCL10, ADAR
hsa05203	Viral carcinogenesis	17	0.02	0.03	HPN, YWHAZ, HIST1H2BC, HIST1H2BD, SP100, UBE3A, GTF2H4, CDK6, CCNE2, CCND1, KRAS, CDKN2A, HIST2H2BE, IRF7, HDAC8, TRAF5, DLG1
**Hypermethylation and low expression**					
hsa04015	Rap1 signaling pathway	16	0.03	9.42E-05	EGFR, FGFR1, MAGI2, FGF7, FLT1, PGF, MRAS, GRIN2A, KIT, CALML3, PDGFRA, RAP1A, GNAS, FGF2, INSR, PIK3R1
hsa04014	Ras signaling pathway	16	0.03	2.13E-04	EGFR, FGFR1, PLD1, FGF7, FLT1, PGF, RALBP1, MRAS, GRIN2A, KIT, CALML3, PDGFRA, RAP1A, FGF2, INSR, PIK3R1
hsa05200	Pathways in cancer	22	0.04	2.55E-04	EGFR, FGFR1, FGF7, WNT5B, PGF, RALBP1, STAT5A, STAT5B, RUNX1T1, KIT, DAPK2, LAMA2, LAMA1, EDNRB, BCL2, PDGFRA, JAK1, NKX3-1, GNAS, FGF2, PIK3R1, APC
hsa04510	Focal adhesion	14	0.02	9.15E-04	EGFR, FLT1, PGF, ITGA1, ITGA10, MYL9, LAMA2, LAMA1, BCL2, PDGFRA, RAP1A, RELN, MYLK, PIK3R1
hsa04151	PI3K-Akt signaling pathway	19	0.03	9.56E-04	EGFR, FGFR1, FLT1, SGK2, FGF7, PGF, ITGA1, ITGA10, KIT, LAMA2, LAMA1, TSC1, BCL2, PDGFRA, JAK1, RELN, FGF2, INSR, PIK3R1
hsa04810	Regulation of actin cytoskeleton	14	0.02	1.09E-03	EGFR, FGFR1, FGF7, ARHGEF7, MRAS, ITGA1, ITGA10, MYL9, PDGFRA, MSN, FGF2, MYLK, PIK3R1, APC
hsa04640	Hematopoietic cell lineage	8	0.01	3.84E-03	CD9, CR1, CD59, ITGA1, MME, KIT, IL11RA, IL1A
hsa05230	Central carbon metabolism in cancer	6	0.01	0.02	EGFR, NTRK3, FGFR1, PDGFRA, KIT, PIK3R1
hsa04270	Vascular smooth muscle contraction	8	0.01	0.02	ACTG2, CALML3, ACTA2, ADRA1A, GNAS, MYLK, PPP1R14A, MYL9
hsa04610	Complement and coagulation cascades	6	0.01	0.02	CR1, A2M, C3, F3, CD59, SERPING1

**Table 3:** Expression validation of 12 upregulated-hypomethylation and 4 downregulated-promotor hypermethylation hub genes by UALCAN.

Category	Hubgenes	Gene description	Degree	Expression status	P value
**Hypomethylation and high expression**	TOP2A	topoisomerase (DNA) II alpha	76	Up-regulated	<1E-12
	MAD2L1	MAD2 mitotic arrest deficient-like 1 (yeast)	38	Up-regulated	1.62E-12
	FEN1	flap structure-specific endonuclease 1	35	Up-regulated	1.62E-12
	EPRS	glutamyl-prolyl-tRNA synthetase	35	Up-regulated	<1E-12
	EXO1	exonuclease 1	33	Up-regulated	<1E-12
	MCM4	minichromosome maintenance complex component 4	32	Up-regulated	1.62E-12
	PTTG1	pituitary tumor-transforming 1	30	Up-regulated	1.62E-12
	RRM2	ribonucleotide reductase regulatory subunit M2	29	Up-regulated	<1E-12
	PSMD14	proteasome 26S subunit, non-ATPase 14	28	Up-regulated	<1E-12
	CDKN3	cyclin dependent kinase inhibitor 3	28	Up-regulated	1.62E-12
	H2AFZ	H2A histone family member Z	27	Up-regulated	<1E-12
	CCNE2	cyclin E2	26	Up-regulated	1.10E-16
**Hypermethylation and low expression**	EGFR	epidermal growth factor receptor	37	Down-regulated	2.20E-16
	FGF2	fibroblast growth factor 2	34	Down-regulated	<1E-12
	BCL2	BCL2, apoptosis regulator	29	Down-regulated	5.40E-04
	PIK3R1	phosphoinositide-3-kinase regulatory subunit 1	27	Down-regulated	1.62E-12

**Table 4:** Biological function of modules identified by Cytoscape.

Category	Module	Function description	P Value	Nodes	Genes
Hypomethylation and high expression					
	1	Cell division	3.30E-06	22	EXO1, RAD51AP1, SHCBP1, PBK, TTK, MCM4, KIF14, ECT2, RRM2, PTTG1, NEK2, CENPF, CDKN3, MAD2L1, UBE2T, E2F8, DTL, CCNE2, ZWINT, FEN1, ASPM, TOP2A
	2	Negative regulation of ubiquitin-protein ligase activity involved in mitotic cell cycle	3.80E-13	10	PSMB5, BUB3, UCHL5, PSMD4, PSMC2, SHFM1, PSMD1, STAMBP, PSMD14, PSMB4
	3	mRNA splicing, via spliceosome	4.20E-07	25	POLD1, PQBP1, PUSL1, MYLIP, H2AFZ, POLR2K, UBE2Q1, UBE2H, U2AF2, CDC5L, NBN, MRE11A, EPRS, TCEB1, SOCS1, SF3B4, RPL7, RBM8A, RSRC1, RAD54B, FBXO22, SNRPB, FBXO6, BRIP1, SEC61A1
	4	tRNA aminoacylation for protein translation	5.00E-08	5	GARS, TARS, DARS2, CCT5, IARS2
	5	Mitochondrial translational elongation	6.60E-17	8	MRPS5, MRPS14, MRPL42, GFM2, CHCHD1, MRPS34, MRPL24, MRPL19
Hypermethylation and low expression					
	1	Mesenchyme migration	2.40E-03	9	ITGA1, MYL9, MYLK, ACTG2, TPM2, CNN1, ACTA2, LMOD1, TAGLN
	2	Protein ubiquitination	3.60E-06	7	CUL3, ANAPC4, KLHL21, FBXL5, FBXO2, TRIM9, HUWE1
	3	Negative regulation of phosphorylation	4.80E-03	5	CDKN1C, GRB10, PEG3, MEST, PLAGL1
