# Supplementary material for: Significant prognostic values of differentially expressed-aberrantly methylated hub genes in breast cancer
Source: J Cancer. 2019 Oct 21;10(26):6618–34. doi: 10.7150/jca.33433 (PMC6856906; doi:10.7150/jca.33433)
Supplement: Supplementary file 1 — Supplementary figures and tables. [file jcav10p6618s1.pdf]

Supplementary Figure legend

Figure S1. PPI network of upregulated-hypomethylated genes and five modules identified by Cytoscape. (A) PPI network; (B-F) Modules identified by Cytoscape

Figure S2. PPI network of downregulated-hypermethylated genes and three modules identified by Cytoscape. (A) PPI network; (B-D) Modules identified by Cytoscape

Figure S3. Expression validation in TCGA database of 16 hub genes (HiSeq data sets (RNASeqV2)). (A-L) Expression of upregulated-hypomethylated hub genes. A: TOP2A; B: MAD2L1; C: FEN1; D: EPRS; E: EXO1; F: MCM4; G: PTTG1; H: RRM2; I: PSMD14; J: CDKN3; K: H2AFZ; L: CCNE2; (M-P) Expression of downregulated-hypermethylated hub genes. M: EGFR; N: FGF2; O: BCL2; P: PIK3R1.

Figure S4. ROC curve analysis for 5-year overall survival and Kaplan Meier-plotter analysis of GSE42568 and GSE65194. A. Roc curve analysis for GSE42568. B. Roc curve analysis for GSE65194. (C-E) Overall survival of GSE42568. C: EXO1; D: MCM4; E: EXO1 and MCM4; (F-H) Overall survival of GSE65194. F: EXO1; G: PTTG1; H: EXO1 and PTTG1. Area under the curve (AUC) was listed in parentheses.

Figure S1.

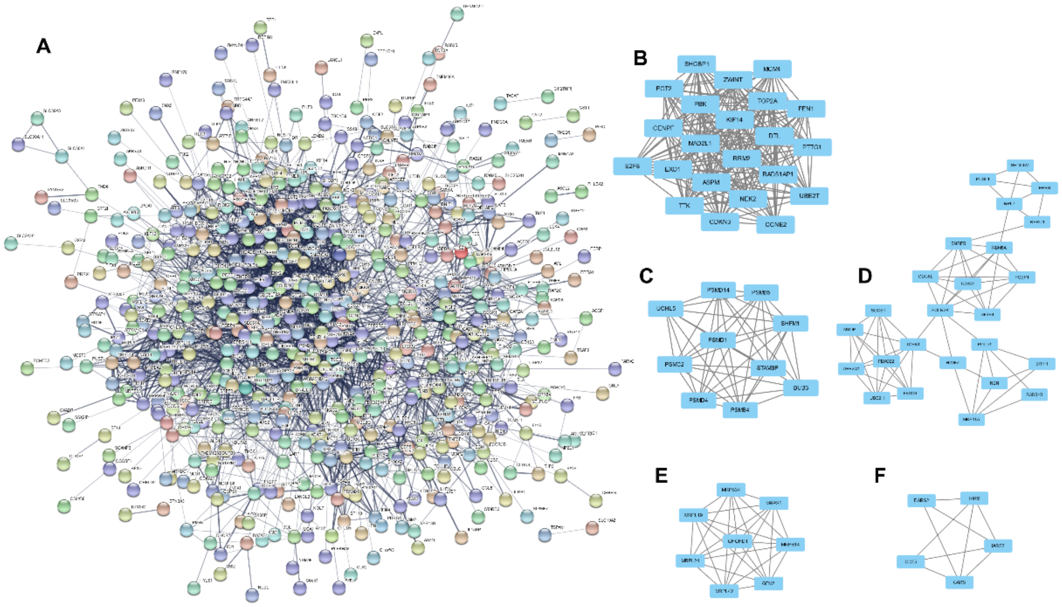

Figure S2.

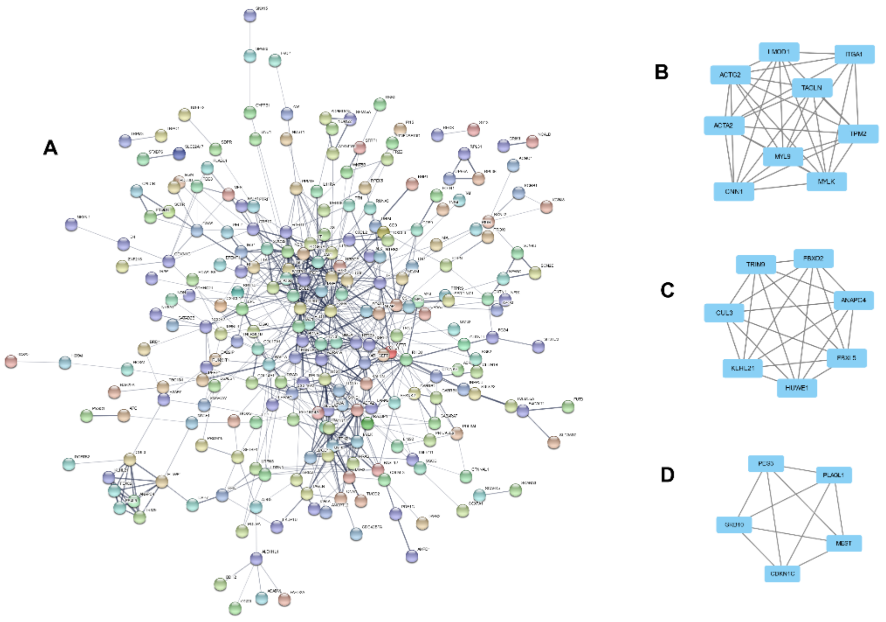

Figure S3.

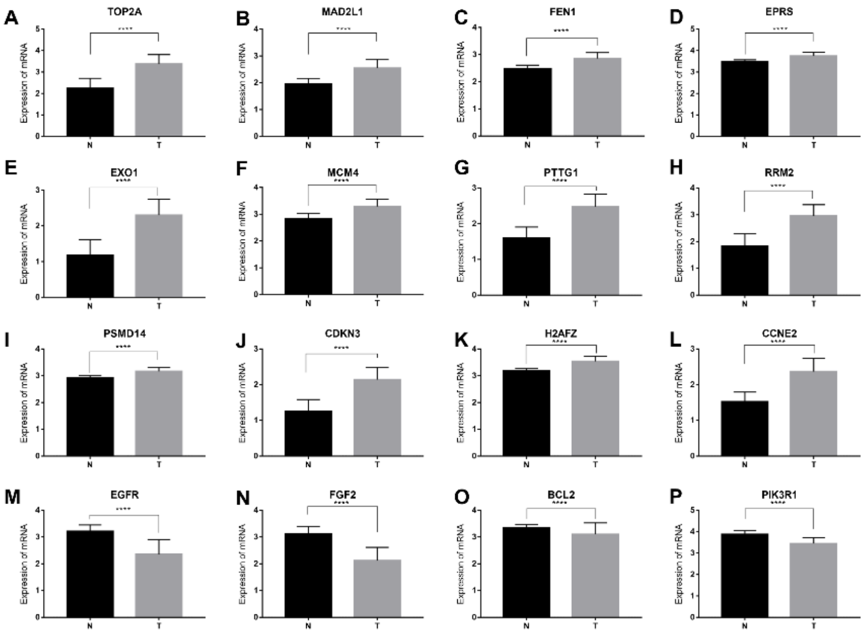

Figure S4.

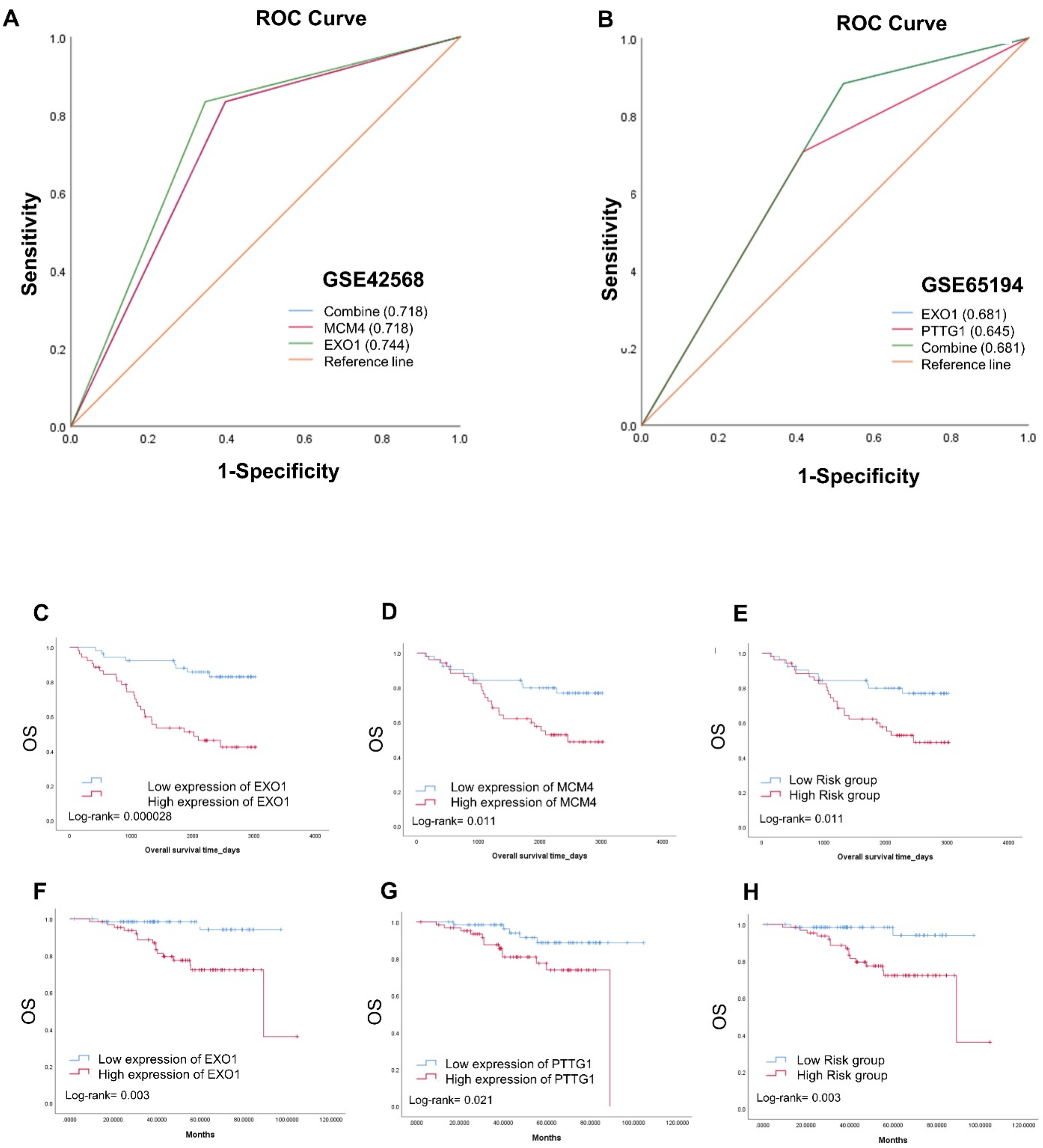
A
ROC Curve
Sensitivity
1-Specificity
GSE42568
Combine (0.718)
MCM4 (0.718)
EXO1 (0.744)
Reference line
B
ROC Curve
Sensitivity
1-Specificity
GSE65194
EXO1 (0.681)
PTTG1 (0.645)
Combine (0.681)
Reference line
C
OS
Low expression of EXO1
High expression of EXO1
Log-rank= 0.000028
Overall survival time_days
D
OS
Low expression of MCM4
High expression of MCM4
Log-rank= 0.011
Overall survival time_days
E
OS
Low Risk group
High Risk group
Log-rank= 0.011
Overall survival time_days
F
OS
Low expression of EXO1
High expression of EXO1
Log-rank= 0.003
Months
G
OS
Low expression of PTTG1
High expression of PTTG1
Log-rank= 0.021
Months
H
OS
Low Risk group
High Risk group
Log-rank= 0.003
Months

Table S1. Univariable and multivariable COX analysis of GSE42568 and GSE65194 dataset s.

		p value	RR	lower 95% CI	upper 95% CI			p value	RR	lower 95% CI	upper 95% CI
	Univariable cox analysis						Univariable cox analysis				
GSE42568	TOP2A	.098	1.219	.964	1.542	GSE65194	TOP2A	.092	1.267	.962	1.669
	MAD2L1	.019	1.442	1.063	1.956		MAD2L1	.626	1.237	.526	2.911
	FEN1	.358	1.199	.814	1.767		FEN1	.062	1.608	.977	2.648
	EPRS	.182	1.386	.858	2.238		EPRS	.105	1.772	.888	3.538
	EXO1	.000	3.593	2.050	6.296		EXO1	.011	1.649	1.119	2.429
	MCM4	.003	1.860	1.229	2.813		MCM4	.063	1.346	.984	1.840
	PTTG1	.011	1.466	1.092	1.967		PTTG1	.045	3.007	1.024	8.829
	RRM2	.649	1.061	.824	1.366		RRM2	.087	1.397	.953	2.047
	PSMD14	.583	1.133	.725	1.769		PSMD14	.357	1.453	.657	3.217
	CDKN3	.014	1.374	1.066	1.772		CDKN3	.273	1.147	.897	1.466
	H2AFZ	.975	1.007	.668	1.518		H2AFZ	.569	1.200	.641	2.249
	CCNE2	.052	1.294	.998	1.678		CCNE2	.032	1.416	1.031	1.945

EGFR	.733	.952	.720	1.260	EGFR	.578	.909	.650	1.272
FGF2	.980	.993	.586	1.684	FGF2	.102	1.281	.952	1.722
BCL2	.570	.066	.000	763.338	BCL2	.825	.919	.435	1.942
PIK3R1	.712	.910	.550	1.504	PIK3R1	.012	.750	.598	.940
**Multivariable cox analysis**					**Multivariable cox analysis**				
EXO1	.001	2.847	1.539	5.270	EXO1	.005	1.862	1.206	2.875
MCM4	.077	1.536	.955	2.471	PTTG1	.014	4.156	1.332	12.967

Table S2. Clinicopathological characteristics of EXO1-expression cohorts in tumor tissue

	EXO1		*P-value*		EXO1		*P-value*
	Low (n=69)	High (n=64)			Low (n=69)	High (n=64)	
Age, years			0.603	AJCC stage			0.681
>50	41	35		1	15	17	
≤50	28	29		2	33	26	
Laterality			0.489	3	21	21	

Left	33	35		ER status			0.643
Right	36	29		Negative	20	19	
T stage			0.559	Positive	46	44	
T1	25	29		Unknown	3	1	
T2	43	34		PR status			0.691
T3	1	1		Negative	30	32	
N stage			0.596	Positive	37	31	
N0	37	35		Unknown	2	1	
N1	11	8		HER2 status			0.067
N2	19	16		Negative	50	56	
N3	2	5		Positive	17	8	
				Unknown	2	0	
